# Versatile Nutraceutical Potentials of Watermelon—A Modest Fruit Loaded with Pharmaceutically Valuable Phytochemicals

**DOI:** 10.3390/molecules25225258

**Published:** 2020-11-11

**Authors:** Abinaya Manivannan, Eun-Su Lee, Koeun Han, Hye-Eun Lee, Do-Sun Kim

**Affiliations:** Vegetable Research Division, National Institute of Horticultural and Herbal Science, Rural Development Administration, Jeonju 55365, Korea; abinaya@korea.kr (A.M.); lus4434@korea.kr (E.-S.L.); hke1221@korea.kr (K.H.); helee72@korea.kr (H.-E.L.)

**Keywords:** anti-cancer, anti-diabetic, functional food, l-citrulline, lycopene, obesity, polyphenols

## Abstract

Watermelon (*Citrulus lantus*) is an important horticultural crop which belongs to the Curcubitaceae family. The nutraceutical potential of watermelon has been illustrated by several researchers, which makes it a better choice of functional food. Watermelon has been used to treat various ailments, such as cardio-vascular diseases, aging related ailments, obesity, diabetes, ulcers, and various types of cancers. The medicinal properties of watermelon are attributed by the presence of important phytochemicals with pharmaceutical values such as lycopene, citrulline, and other polyphenolic compounds. Watermelon acts as vital source of l-citrulline, a neutral-alpha amino acid which is the precursor of l-arginine, an essential amino acid necessary for protein synthesis. Supplementation of l-citrulline and lycopene displayed numerous health benefits in in vitro and in vivo studies. Similarly, the dietary intake of watermelon has proven benefits as functional food in humans for weight management. Apart from the fruits, the extracts prepared from the seeds, sprouts, and leaves also evidenced medicinal properties. The present review provides a comprehensive overview of benefits of watermelon for the treatment of various ailments.

## 1. Introduction

Consumption of fruits and vegetables in regular diet provides several health benefits. The wide occurrence of phytochemicals such as carotenoids, lycopenes, anthocyanins, phenols, and flavonoids along with vitamins and minerals makes the choice of plant based diet healthier. It lowers the risks of various dreadful diseases such as cancer, cardiovascular diseases, neurodegenerative disorders and aging associated ailments. Plants, being the wide source of pharmaceutically valuable secondary metabolites, provide diverse products in the form of fruits and vegetables loaded with nutraceutical potential. In general, the major role of secondary metabolites in plants are pertained to the protection against diverse abiotic and biotic stresses. In addition, secondary metabolites also acts as an antimicrobial agents and antioxidants to combat stresses.

Watermelon is a notable horticultural crop belonging to the Cucurbitaceae family cultivated widely for its delicious fruits. Asian countries contribute approximately 81% of total production of watermelon worldwide [1]. According to the Food and Agricultural Organization of the United Nations, a cultivation area of 3.2 million hectares was employed for the production of 103 million tons of watermelon worldwide in 2018 [1]. The watermelon fruits are used for the preparation of smoothies, jams, sauces, candies, and juices. Watermelon serves as a vital natural source of l-citrulline (0.9 to 5 mg/kg of fresh fruit) [2]. The refreshing taste, high water content, and its attractive colors ranging from red, yellow, and pink increases the consumption of watermelon. The diverse colors of watermelon are due to the presence of carotenoids especially, lycopene and β-carotene [2]. The sweet taste of watermelon is contributed by the combination of sugars such as glucose, sucrose, and fructose. Moreover, watermelon, acts as a vital reservoir of valuable phytochemicals with high nutrition and pharmaceutical potentials. In particular, watermelon can be considered as an excellent functional food due to its rich lycopene, vitamin A, vitamin C contents and antioxidant potentials [3,4]. Bioactive compounds present in watermelon render numerous health benefits, such as decreasing the risk of cardio-vascular disease, aging related ailments, obesity, diabetes, and various cancer alleviating effects have been reported [5,6,7,8,9,10]. In 1930, Wada [11] determined and isolated citrulline, a non-essential amino acid from watermelon which is involved in the synthesis of arginine. The amino acid arginine is vital for the endogenous synthesis of nitric oxide, a crucial signaling molecule involved in various neurological and immune responses in animals and humans [12]. Watermelon acts as a vital dietary supplement to enhance the arginine content. In watermelon, citrulline aid in the tolerance against stresses such as drought [13]. Moreover, the seeds of watermelon are enriched with protein, fat, and moderate levels of iron and zinc. Watermelon seeds are consumed as snacks, fat binder, soup thickener, condiments, and also for the extraction of cooking oil [14,15,16]. The occurrence of high arginine content in the seeds of watermelon adds its medicinal benefits [17]. Due to the presence of various nutritious benefits the seeds of watermelon possess application in the field of several food products. Recently, Sola et al. [18] identified and quantified the phytochemicals in the methanol extracts derived from the seeds of watermelon. Furthermore, the report [18] evidenced the anti-bacterial property of watermelon seed extracts.

The present review deals with various nutraceutical potentials of watermelon and its importance as an antioxidant, anti-cancerous, cardiovascular protectant, anti-inflammatory properties evidenced by in vitro and in vivo studies.

## 2. Cardiovascular Protection by Watermelon

Cardiovascular diseases are the leading cause of increasing death rate worldwide. Moreover, the cost of treating cardiovascular disease are high. Therefore, adapting a lifestyle with cardio-friendly diet would decrease the risk factors associated with the disease. Fruits and vegetables can combat the negative effects of cardiovascular diseases. l-citrulline and l-arginine possess the capacity to alleviate the inflammation and oxidative stress [19,20]. However, direct intake of l-citrulline and l-arginine could lead to gastro-intestinal discomforts such as nausea and diarrhea [21,22]. Therefore, the consumption of fruits rich in l-citrulline (precursor of l-arginine, an essential amino acid for protein synthesis)—such as watermelon—is important to obtaining the necessary nutrition. Supplementation of whole watermelon in powder form improved the lipid profiles, antioxidant status, and anti-inflammatory properties of high fat fed rats [23]. Moreover, the ingestion of watermelon regulated the expression of genes associated with lipid metabolism [23]. In detail, the augmentation of watermelon and l-arginine enhanced the regulation of hepatic gene expression of endothelial nitric oxide synthase. Nitric oxide (NO) is a ubiquitous signaling molecule vital for the relaxation of blood vessels and it also reduces the atherosclerosis by influencing the lipid metabolism [23,24,25]. On the other hand, watermelon supplementation down-regulated the genes involved in lipid metabolism such as fatty acid synthase (FAS), 3-hydroxy-3methyl glutaryl-coA reductase (HMGCR), sterol regulatory element binding protein (SERB) 1, SERB 2, cyclooxygenase-2 (COX2), and nuclear factor-kB (NF-κB) in rats [23]. Among the above enzymes, FAS plays an important role in the denovo synthesis of fatty acids, whereas HMGCR acts as a rate limiting enzyme in cholesterol synthesis [26]. Similarly, both SREBP-1 and SREBP-2 regulate the transcription of genes involved in fatty acid and cholesterol synthesis respectively [27]. 

Oxidative stress and inflammation are the key players in the onset of atherosclerosis. The serum C-reactive protein levels are utilized as the indicators of systemic inflammation which leads to cardiac dysfunction [28,29]. Watermelon intake significantly reduced the levels of C-reactive protein levels in the serum of high fat fed rats [30]. Moreover, watermelon also down-regulated the expression of Cox-2 enzyme responsible for the synthesis of pro-inflammatory prostaglandins. Furthermore, Hong et al. [30], illustrated that the watermelon supplementation exhibited similar mechanism to non-steroidal anti-inflammatory drugs that inhibits the activity of Cox-2 and reduces the inflammatory response. A recent study has demonstrated the ability of watermelon to reduce the risk factors of cardiovascular disorder in human [31,32]. According to Connolly et al. [32], consumption of watermelon in daily basis for a period of four weeks resulted in significant reductions in body weight, body mass index, waist-to-hip ratio, and blood pressure. In addition, the report also claims that the watermelon supplementation lowered the levels of triglyceride, low density lipoprotein cholesterol, thiobarbituic acid reactive substance, and increased the antioxidant capacity in obese adults [32]. Overall, it has been evident that the consumption of watermelon in regular basis reduces the risk factors associated with chronic illnesses such as cardiovascular diseases.

## 3. Watermelon as a Functional Food in Obesity Management and Anti-Diabetic Snack

Obesity is an alarming public health issue worldwide which is linked to crucial metabolic ailments including diabetes and lifestyle-related diseases. Modern lifestyle and un-healthy food habits including several fast foods and processed food with higher levels of sugar in routine diet are major contributing factors for obesity. According to the National Diabetics Statistics Report (2020) published by Centers for Disease Control and Prevention (CDC), 45.8% of adults in USA are obese, among which 10.5% of the population suffer with diabetes [33]. Diabetes can be classified into two types (1 and 2) depending on the etio-pathogenesis. Type 1 diabetes is characterized by the destruction of pancreatic B cells due to the autoimmune response of the body leading to the insulin deficiency, whereas type 2, the most common form of diabetes involving resistance to insulin [34]. The chronic hyperglycemia resulting from the diabetes leads to retinopathy, neuropathy, nephropathy, peripheral vascular diseases, cerebrovascular diseases, and ischemic heart diseases [35,36,37]. However, the oxidative stress level and inflammatory responses in the body play a vital role in the development of the abovementioned complications.

The prominent symptom of non-insulin-dependent diabetes is the reduction in endothelial NO synthesis and bioavailability, increase in glucose concentration in plasma, free fatty acid, homocysteine, and methylarginines [38]. Numerous studies suggest that the regulation of energy substrate oxidation, sensitivity to insulin and hemodynamics are influenced by NO in humans [24]. For the synthesis of NO, l-arginine acts as the precursor which is converted by tetrahydrobiopetrin (BH_4_)-dependent NO synthase [9]. Based on the results of various in vivo and in vitro studies, it has been demonstrated that the dietary intake of l-arginine decreased the glucose level in diabetic and obese rats [39,40,41]. In addition, the l-arginine enhanced the vascular reactivity in animal models [38,42,43] and hypercholesterol patients [44]. Thus, owing to the numerous health benefits of l-arginine, it has been recommended to alleviate the risk of obesity and diabetes. However, direct intake of l-arginine displayed gastro-intestinal problems therefore diets enriched with l-arginine have become the alternative. 

Consumption of watermelon significantly increased the plasma l-arginine levels. According to Wu et al. [12], supplementation of watermelon juice in Zucker diabetic fatty rats (a widely used animal model for non-insulin dependent diabetes) elevated the l-arginine levels, decreased the amount of glucose, free fatty acid, homocysteine, and methylarginines [12]. On the other hand, it enhanced the activity of GTP cyclohydroxylase-1 and tetrahydrobiopterin levels in heart and acetylcholine-mediated vascular relaxation. In addition, Wu et al. [12] recommended the consumption of watermelon pomace juice as a functional food to combat diabetes and obesity based on their results in animal models. 

In humans, the oral administration of watermelon juice can act as an effective alternative for the supplementation of arginine. Watermelon juice effectively aided in the regulation of whole body metabolism, and enhanced the cardiovascular and immune response in humans. A study by Figueroa et al. [45] in obese middle-aged adults with pre-hypertension revealed that the watermelon intake improved the arterial function and reduced the ankle blood pressure, brachial blood pressure, and carotid wave reflection. Similarly, consumption of watermelon elicited the satiety response, and reduced the body weight, body mass index (BMI), and waist to hip ratio in obese adults. Furthermore, Lum et al. [46] suggested that watermelon can effectively reduce the appetite and aid in the weight management on comparison to the conventional refined carbohydrate snacks. 

## 4. Anti-Ulcerative Colitis Property of Watermelon 

Ulcerative colitis is one of the inflammatory bowel disease occurring broadly which causes the mucosal inflammation in the entire bowel system [47]. The characteristic feature of ulcerative colitis is the destruction of goblet cells, crypts, and development of ulcers [48]. In addition, ulcerative colitis in the chronic stages can develop into the deadly colorectal cancer, which is the second leading cancer with high death rate [49]. Apart from colorectal cancer, ulcerative colitis is also associated with the onset of other related ailments such as rheumatoid arthritis, ankylosing spondylitis, and psoriasis [50]. The reduction in the absorption of l-arginine by colonocytes is one of the symptoms of ulcerative colitis [51,52]. According to Coburn et al., [53] the patients with ulcerative colitis displayed lesser content of l-arginine which influenced the histology of colon and normal mucosal permeability. Furthermore, the supplementation of l-arginine alleviated the symptoms of ulcerative colitis by increasing the activity of antioxidants, lowering the pro-inflammatory cytokine levels, and also by improving other allied clinical parameters [54,55]. The abundance of l-citrulline, precursor of l-arginine in the watermelon can aid in the treatment of ulcerative colitis. A recent study by Hong et al. [30] demonstrated that the watermelon supplementation mediated improvement of the micro-architecture of colon crypts, cellular kinetics, and an increase in the endogenous levels of nitric oxide. Hong et al. [30] hypothesized that the upregulation of NO levels by watermelon would synergistically enhance the expression of peroxisome proliferator-activated receptor–γ (PPAR-γ) which in turn alleviated the inflammation and oxidative stress. Previous studies suggested the direct influence of NO and l-arginine on the up-regulation of PPAR-γ whereas ulcerative colitis leads to the reduction in the levels of PPAR-γ [56,57]. The important enzymes such as COX-2, iNOS, and NF-κB involved in the generation of reactive oxygen species are inhibited by PPAR-γ; on the other hand, the activities of antioxidant enzymes are enhanced by PPAR-γ [58]. 

In addition, the watermelon augmentation decreased the levels on cyclin D1, a vital protein in the Wnt signaling pathway in the high fat fed mice with ulcerative colitis [30]. Elevated expression of cyclin D1 is widely observed in ulcerative colitis and colon carcinogenesis [59]. The Wnt signaling pathway triggers the cellular proliferation rather than cellular differentiation, leading to carcinogenesis [59]. Previous reports suggested the endogenous NO mediated inhibition of Cyclin D1 regulation via β-catenin pathway [59,60], therefore the increase in the NO level by watermelon could decrease the expression of Cyclin D1. 

One of the major factor that increases the pathogenesis of ulcerative colitis is the generation of excess reactive oxygen species resulting in oxidative stress which is lethal to the macromolecular components of the cells and also leads to DNA damage [61]. In particular, the oxidative stress causing DNA damages leads to the promotion of carcinogenesis. For instance, the generation of 8-hydroxydeoxyguanosine (8-OHdG), an oxidized derivative of deoxyguanosine formed by the reaction between reactive hydroxyl radical and DNA nucleobase facilitates the onset of colorectal cancer via the down-regulation of tumor suppressor genes [62,63]. Hong et al. [30] have identified the elevated levels of 8-OHdG in rats treated with DSS, whereas supplementation of watermelon reduced the 8-OHdG content. The abundant antioxidant property of watermelon can be responsible for the suppression of 8-OHdG levels by alleviating the oxidative stress and thus protecting the DNA from damage. 

## 5. Anti-Oxidant Properties of Watermelon

Several cascades of biochemical reactions are continuously undergoing in the cells and organelles of a human body. The constant metabolic reactions generate highly reactive free radicals in the system as inevitable by-products. These free radicals have the tendency to react with the macromolecules such as proteins, lipids, and also with the DNA molecules and affects their normal functions [64]. The cellular redox reactions generate reactive oxygen species which can act as beneficial secondary signaling molecules, but in excess cause oxidative stress in the cells. The oxidative stress potentially damages the cellular organelles, which in the long term can result in various life-threatening ailments, such as cancers, cardiovascular, and other neurodegenerative diseases [64]. However, diets enriched with antioxidants can prevent the oxidative damages caused by the ROS. Plant-based diets are the major source of antioxidants. In particular, phytochemicals such as phenols, flavonoids, alkaloids, and terpenoids are the vital antioxidant molecules with several nutraceutical benefits [65]. These phytochemicals are termed as secondary metabolites which are observed in lower abundance in plants in comparison with primary metabolites and distributed in specialized cells or organelles [66]. Secondary metabolites plays a vital role in the interaction between the plant and environment. It aids in the protection against abiotic and biotic stresses in plants and also provides colors and aroma to plants and plant products [66].

The primary antioxidant potential of a secondary metabolite is acclaimed by the ability of the compound to detoxify the toxic ROS by scavenging or by the prevention of oxidation of low-density lipoproteins. In recent times, various health benefits rendered by plant-based polyphenolic compounds have become the focus of several researchers. Similarly, pigments such as lycopene and β-carotene present in watermelon also displayed antioxidant properties. Reports suggested that the consumption of lycopene and β-carotene protects the architecture of plasma lipoprotein from oxidative stress, suppresses the macular degeneration, prevents cataracts, and decreases the bioavailability of nitrogen oxide [67,68,69]. In addition, the pigments aided in the improvement of immune system and prevented the progression of tumors [70]. Among the carotenoids, lycopene consists of strong antioxidants, for instance, the free radical scavenging rate of lycopene is higher in comparison with carotenoids such as β-carotene and tocopherol. According to previous reports, the capability of lycopene to quench the singlet oxygen is ten times higher than tocopherol and two-fold higher than β-carotene [71,72]. Among the fruits, watermelon consists of higher contents of bioavailable lycopene (1:12 of carotene) followed by tomato [70]. However, the content of lycopene differs among the different cultivars of watermelon as well as being determined by the growing environment [72,73]. The abundance of lycopene in watermelon makes it as an excellent choice of functional food. The consumption of a lycopene-rich diet can effectively improve the detoxification of free radicals which pose a threat to DNA and cellular membrane and also regulates the lipid biosynthesis by influencing the enzymes in the cholesterol biosynthesis pathway [74,75,76]. 

Polyphenolic compounds are vital antioxidants classified into phenolic acids, flavonoids, lignans, and stilbenes. The polyphenol structure contains a minimum of two hydroxyl groups attached to an aromatic ring. In fruits and vegetables, the majority of the antioxidant properties are attributed by the polyphenolic compounds. According to Tilili et al. [7], in watermelon, the occurrence of polyphenols is responsible for the hydrophilic antioxidant activity, and the fresh juice of watermelon is reported to have 16.94–20.23 mg Gallic acid equivalent (GAE)/100 mL of polyphenols. Therefore, the intake of watermelon as a dietary snack or in beverage form can induce the antioxidant potentials in the human body and helps in the improvement of cell signaling, adhesion, and other biological activities. However, the structure, level, absorption, and bioavailability of polyphenols determine the antioxidant potentials [77]. Several polyphenols have been encompassed in the watermelon and few studies have attempted to identify the polyphenolic contents (Table 1). Abu-Reidah et al. [78], have characterized phytochemicals present in the methanolic extracts of watermelon using high-performance liquid chromatography coupled to electrospray ionization–quadrupole time-of-flight mass spectrometry (HPLC–ESI–QTOF–MS). 

Apart from the direct consumption, watermelon can be added into food products, for instance the augmentation of watermelon rind powders as a component in cake enhanced the free radical scavenging activity and beta-carotene levels. Supplementation of watermelon rind powder along with wheat flour during the preparation of cake increased the moisture, fat, protein, and carbohydrate contents [79]. In addition, the watermelon rind powder consisted of different polyphenolic substances such as 4-hydroxybenzoic acid, vanillin, and coumaric acid [79]. The presence of polyphenolic compounds in watermelon rind powder significantly increased the efficiency of 1, 1-diphenyl-2-picryIhydrazyl (DPPH) radical scavenging [79]. The antioxidant capacity resulting from the addition of watermelon rind in the cakes can also improve the shelf life along with the enhancement of the functional components like polyphenols. Similarly, the inhibition of DPPH radicals by watermelon extracts was reported by Tita et al. [80]. The report has analyzed the antioxidant ability and other health promoting biological functions of pulp and rind extracts of various watermelon cultivars. Similarly, the study led by Choudhary et al. [81], evidenced the presence of health promoting phytochemicals and antioxidant potentials of various watermelon cultivars using in vitro assays. The results suggested that the mean antioxidant activity determined using cupric reducing antioxidant capacity assay of different watermelon cultivars ranged between 40.13 to 84.05 μmol Trolox equivalents (TE)/100 g FW. Moreover, the report also estimated the phytochemicals such as total phenol (16.77 to 21.41 mg/g DW), total flavonoids (55.60 to 100.93 mg/100 g DW), tannin (35.07 to 60.83 mg/100 g DW) and carotenoids (4.90 to 8.06 mg/100 g), and lycopenes (3.74 to 6.80 mg/100 g) with well-known antioxidant ability [81]. According to the results observed in different cultivars, a higher amount of phytochemicals with antioxidant capacity has been determined in the red-fleshed watermelons [81]. Similarly, the red fleshed watermelon consisted of higher levels of ascorbic acid (86.32 mg/kg) and lycopene (9.50 mg/kg) in comparison with the yellow fleshed watermelon (ascorbic acid: 52.05 mg/kg; lycopene: (0.04 mg/kg), which correlated with the antioxidant capacity [82]. The antioxidant capacity of the watermelon has been evaluated using the DPPH radical scavenging potential and ferrous ion chelating activity. 

In addition to the rind and flesh, watermelon peels also exhibited the antioxidant capacity. The investigation of phytochemicals and mineral components in the rind and peel of watermelon can promote the utilization of low-cost agricultural by products for commercial use in small and large scale food and allied industries. For instance, the report by Feizy et al. [83] has determined the presence of protein, fat, ash, fiber, sodium, potassium, calcium, copper, magnesium, iron, phosphorus, and zinc in the watermelon peel. Furthermore, the peel tissue consisted of significant amounts of polyphenols which aided in the scavenging of free radicals. In general, the synthetic antioxidants such as butylated hydroxyanisole (BHA) and butylated hydroxytoluene (BHT) are supplemented to increase the shelf life and stability of the processed food and food products [83]. These synthetic antioxidants consists of several potential disadvantages which needs a natural replacement. Therefore, the application of fruits and vegetable by-products could render a possibility for an effective substitute. Taken together, the consumption of watermelon as both direct dietary supplement or as indirect food supplement can aid in the improvement of antioxidant potentials.

## 6. Anticancer Properties of Watermelon 

Cancer is a dreadful disease with high fatality rate in many nations worldwide. The association between the active components of diet with the expression of genes in several metabolic pathways can influence the molecular mechanism of carcinogenesis in biological system. For instance, the lycopene, an active component in watermelon can indulge in the modulation of cancer development by the inhibition of DNA mutation and acting against the metastasis of the tumor [95]. According to Nahum et al. [95], lycopene induces modulations in the cell cycle machinery, particularly by the inhibition of G1 phase in human breast and endometrial cancer. The administration of lycopene resulted in a reduction in cyclin-dependent kinase (CDK) 1 and 3 activities in cancer cells. Moreover, the antioxidant property of lycopene reduces the oxidative stress and aids in the anti-proliferative effects against cancerous cells. Several studies have demonstrated the anti-cancerous potential of lycopene in vitro and in vivo [96]. However, the in-depth molecular rationale behind the lycopene-mediated regulation of interaction between genes are still in study. 

Among the cancers, the colon cancer is the second major deadliest cancer worldwide affecting humans. Colon cancer can occur due to the disturbance of the balance between the cellular proliferation and the programmed cell death (apoptosis). However, studies suggested that the proper dietary intervention can prevent most of the colon cancers. The supplementation of watermelon in rats with colon cancer decreased the cellular proliferation but no significant effects on apoptosis have been observed [97]. The tumoricidal property of the watermelon can be due to various factors, but the vital one could be the occurrence of abundant l-citrulline and its function in the synthesis of endothelial nitric oxide (NO). According to Glen et al. [98], the addition of watermelon powder in the diets of male Sprague-Dawley rats induced with colon cancer reduced the risks by alleviating the formation of aberrant crypt foci via reducing the oxidative damages and inflammation to DNA. In addition, an increase in the production of endogenous nitric oxide added the alleviation of cancerous effects and the watermelon supplementation also modulated the expression of DNA repair enzymes to combat the cancer. 

In women, breast and cervical cancers are the two most major cancers with high fatality rate. The anti-proliferative effects of watermelon leaf extracts have been investigated on both breast and cervical cancer cell lines [99]. Leaf extracts from six cultivars of watermelon were tested in cervical cancer cell lines (C33A, HeLa and SiHa) and breast adenocarcinoma cell lines (MDA-MB-231 and MCF-7) [99]. The in vitro MTT assay and the microscopic observation of cells evidenced the anti-proliferative property of watermelon leaf extracts in both cancer cell lines on comparison with normal cells. However, the cervical cancer cell lines, in particular the C33A, displayed high sensitivity to the extracts. Among the cancer cell lines, the microscopic observations elucidated the reduction in the number of cells and cellular size in C33A, MCF-7, and MDA-MB-231 lines [99]. The report also suggested the cultivar dependent anti-cancer property of watermelon leaf extracts. 

**Figure 1:**
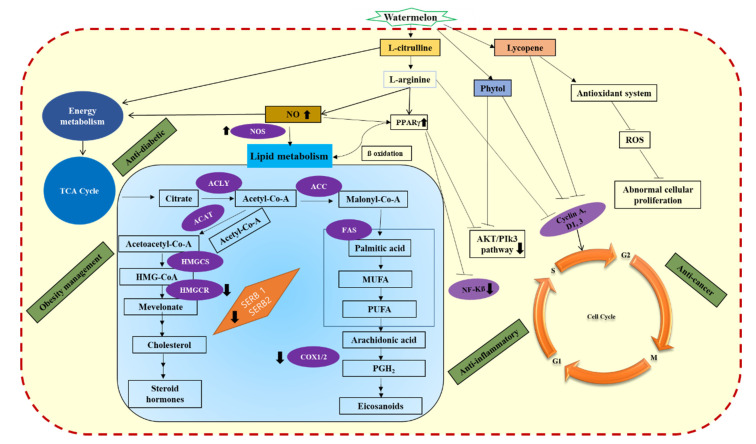
A schematic representation of various mechanisms induced by the phytochemicals present in watermelon extracts. The figure has been conceived based on the interpretation of the literatures cited in sections (2-6). ACC, acetyl-coA carboxylase; ACLY, ATP-citrate lyase; COX1/2,cyclooxygenase; Akt, alpha serine-threonine protein kinase; HMGCS, 3-hydroxy-3-methylglutaryl-CoA synthase; HMGCR, 3-hydroxy-3-methylglutaryl-CoA reductase; FAS, fattyacid synthase; MUFA, monounsaturated fatty acid; NO, endogenous nitric oxide; NOS, nitric oxide synthase; PPAR-γ, peroxisome proliferator-activated receptor–γ; PUFA, polyunsaturated fatty acid; PGH2, prostaglandin-H_2_; ROS, reactive oxygen species, SERB, sterol regulatory element binding proteins; TCA, tricarboxylic acetic acid cycle.

Leukemia is caused by the abnormal proliferation of blood cells in the bone marrow. Various parts of watermelon have the anti-cancer potential due to the presence of the vital pharmaceutically valuable phytochemicals. The phytochemical ‘phytol’ extracted from the sprouts of watermelon inhibited the excessive proliferation of human T-cell leukemia line Jurkat cell and human lung adenocarcinoma epithelial cell line A549—xenograft mice model [100]. In addition, the molecular mechanism behind the phytol-mediated cell death included the activation of intercellular reactive oxygen species via NADPH oxidase, which resulted in the arrest of cell cycle in S-phase. The expressions of vital proteins such as cyclin A, cyclin D, mitogen activated protein kinase (MAPK) and phosphatidylinositol-3-kinase (PI3K)/protein kinase b (Akt) were downregulated which resulted in the S-phase arrest [100]. The protein cyclin A is vital for the synthesis of DNA molecules and for the advancement of S-phase in the cell cycle by binding to Cdk2 protein [101,102]. Furthermore, the transition of G1 to S phase in the cell cycle is mediated by cyclin D; for instance, the cyclin D phosphorylates the retinoblastoma tumor suppressor protein by docking directly to Cdk4 or Cdk6 [103,104]. Moreover, the report suggested that the regulation of cyclin A and D proteins was mediated by the ROS which prevented the cell cycle in S-phase [100]. Similarly, several reports have illustrated the regulation of cyclin A and D expressions mediated by the MAPKs and PI3K/Akt [105,106,107,108,109]. Both MAPKs and PI3K/Akt are involved in numerous functions of cells such as proliferation, movement, differentiation, elongation, survival, growth, and death [110]. However, the apoptosis mechanism was not significantly related to the phytol-induced cell death in the cancer cell lines. Similar observation of non-apoptosis mediated cell death primarily triggered by the intercellular ROS in cancer cell lines by phytochemicals have been reported [111]. Overall, the secondary metabolites present in watermelon tissues can effectively reduce the proliferation of cancer cells. Further studies related to the identification and extraction of the potential phytochemicals with efficient anti-cancer activity can aid in the search for drug candidates for several dreadful cancers. Intake of watermelon displayed potential health benefits against several life-threatening diseases. The phytochemicals present in the different tissues of watermelon combat the harmful oxidative stress and also influenced vital metabolic pathways (Figure 1). Most of the molecules targeted by the phytochemicals in watermelon are involved in diverse metabolic networks, therefore, the molecular rational behind the disease alleviation by watermelon have to be studied in detail by considering the isolation of active components in the extract.

## 7. Conclusions

Watermelon encompasses a diverse amounts of phytochemicals with vital pharmaceutical importance majority of which have been attributed by the abundant occurrence of citrulline, lycopenes, and polyphenols. The supplementation of watermelon extracts under various pathological ailments aids in the convalescence of diseases as well as from their inevitable negative side effects. Secondary metabolites with nutraceutical potential present in different tissues of watermelon—such as leaves, sprouts, seeds, rinds, and fruits—act on different potential drug targets involved in diseases such as diabetes, cancer, inflammation, and obesity. However, further studies dealing with the extraction of the active phytochemical and investigation of molecular regulatory mechanisms of the bioactive compounds in watermelon are required to extend the utilization of the phytochemicals in nutraceutical industries. In addition, the phytochemical and pharmacokinetic interpretation of vital secondary metabolites in watermelons can facilitate the drug designing process to combat dreadful diseases. Moreover, being the functional food intake of watermelon can prevent the onset of various disorders in humans. Taken together, to accelerate the discovery of several plant based drug candidates with minimal side effects, the knowledge gained from the phytochemicals present in the fruits and vegetables can be a valuable asset. 

## Figures and Tables

**Table 1:** Phytochemicals profiled in watermelon with antioxidant property.

S. NO	Compound Name	Class	Reference
1.	3-O-Feruloylsucrose	Hydroxycinnamic acid derivatives	[84]
2.	Ajugol	Iridoid	[78]
3.	Apigenin-*O*-hexoside I	Flavonoid	[78]
4.	Apigenin-*O*-hexoside II	Flavonoid	[78]
5.	Aviprin I	Coumarin	[78]
6.	β-carotene	Carotenoid	[2]
7.	Caffeoylhexose I	Hydroxycinnamic acid derivatives	[78]
8.	Catalposide	Iridoid	[78]
9.	Chrysoeriol-*O*-hexoside I	Flavonoid	[78]
10.	Chrysoeriol-*O*-hexoside II	Flavonoid	[78]
11.	Cimifugin	Phenol	[78]
12.	Citrulline	Amino acid	[4]
13.	Coumarin	Coumarin	[78]
14.	Decaffeoylacetoside	Hydroxycinnamic acid derivatives	[78]
15.	Dicaffeoylshikimic acid II	Hydroxycinnamic acid derivatives	[85]
16.	Dihydrophilonotisflavone	Flavonoid	[78]
17.	Eriodictyol 7-glucoside	Flavonoid	[78]
18.	Ferulic acid hexoside I	Hydroxycinnamic acid derivatives	[78,86]
19.	Glehlinoside C	Phenol	[78]
20.	Hydroquinone glucuronide	Phenol	[78]
21.	Isolariciresinol 9′-β-d-glucopyranoside I	Lignan	[78]
22.	Isoorientin	Flavonoid	[78]
23.	Isovitexin	Flavonoid	[87,88]
24.	Kaempferol rhamnoside–hexoside I	Flavonoid	[78]
25.	Leachianol G	Phenol	[78]
26.	Lucenin-2-methyl ether	Flavonoid	[78]
27.	Luteolin-*O*-hexoside I	Flavonoid	[89]
28.	Lycopene	Carotenoid	[2]
29.	Naringenin 7-rutinoside I	Flavonoid	[78]
30.	*O*-Feruloylquinide	Hydroxycinnamic acid derivatives	[86]
31.	Obtusoside	Coumarin	[78]
32.	p-Coumaric acid glucoside I	Hydroxycinnamic acid derivatives	[78,90]
33.	p-Coumaric acid glucoside II	Hydroxycinnamic acid derivatives	[78,90]
34.	Phloroglucinol glucuronide	Hydroxybenzoic acid derivative	[78]
35.	Picroside	Iridoid	[91]
36.	Protocatechuic acid glucoside I	Hydroxybenzoic acid derivative	[92]
37.	Protocatechuic acid glucoside II	Hydroxybenzoic acid derivative	[92]
38.	Quercitin	Flavonoid	[78]
39.	Rutin	Flavonoid	[78,93]
40.	Salicylic acid-*O*-hexoside I	Hydroxybenzoic acid derivatives	[94]
41.	Salicylic acid-*O*-hexoside II	Hydroxybenzoic acid derivatives	[94]
42.	Saligenin glucopyranoside	Phenol	[78]
43.	Shikonine	Phenol	[78]
44.	Sinapic acid glucoside	Hydroxycinnamic acid derivatives	[78]
45.	Taxifolin-*O*-hexoside I	Flavonoid	[78]
46.	Tri-*O*-caffeoylshikimic acid I	Hydroxybenzoic acid derivatives	[85]
47.	Vanillic acid hexoside	Hydroxybenzoic acid derivatives	[78]
48.	Vanillin hexoside I	Hydroxybenzoic acid derivatives	[78]
49.	Vanillin hexoside II	Hydroxybenzoic acid derivatives	[78]
50.	Vanillin hexoside III	Hydroxybenzoic acid derivatives	[78]
